# Supplementary figures and images for: Comparison of BMIPP-SPECT/CT to 18FDG-PET/CT for Imaging Brown or Browning Fat in a Preclinical Model
Source: Int J Mol Sci. 2022 Apr 28;23(9):4880. doi: 10.3390/ijms23094880 (PMC9101718; doi:10.3390/ijms23094880)

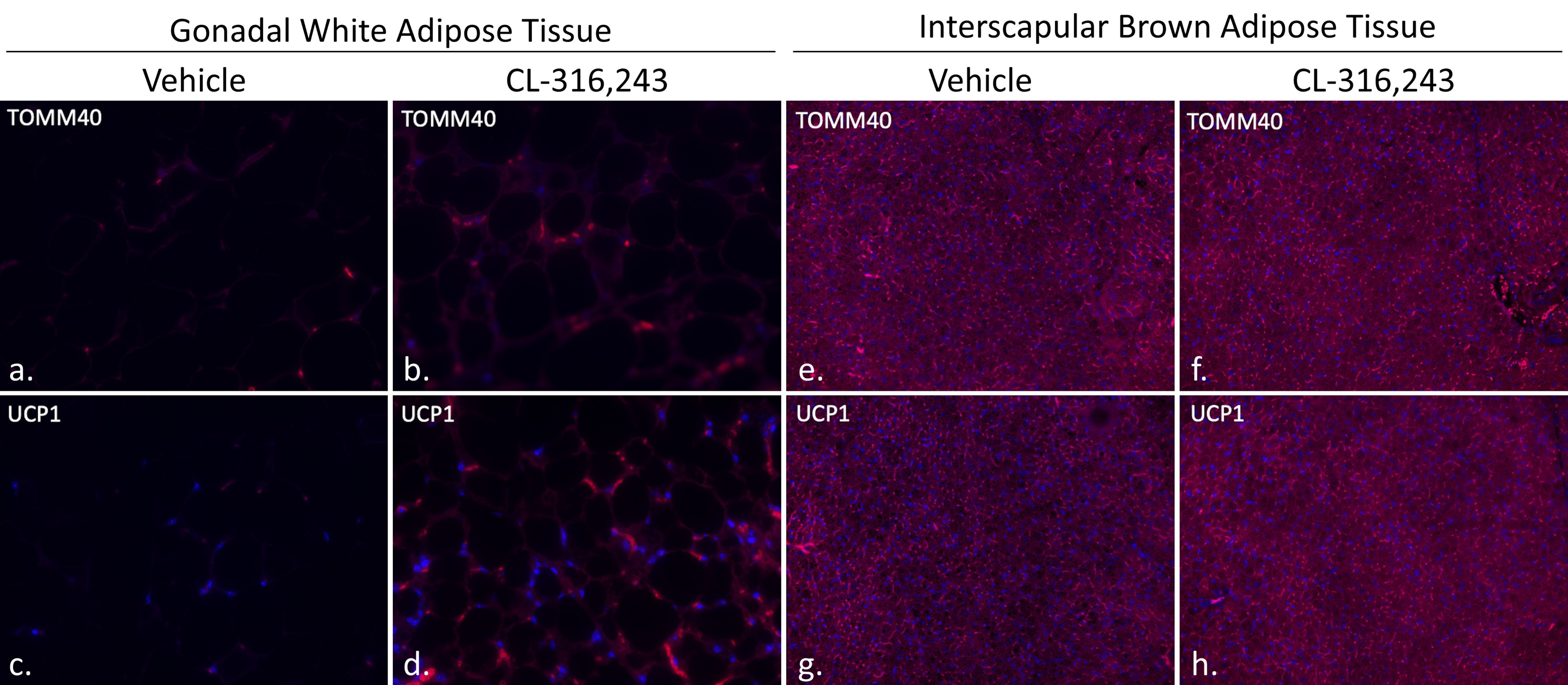

Supplement: Supplementary file 1 [file ijms-23-04880-s001.zip › Figure S1 - UCP1 IF.jpg]

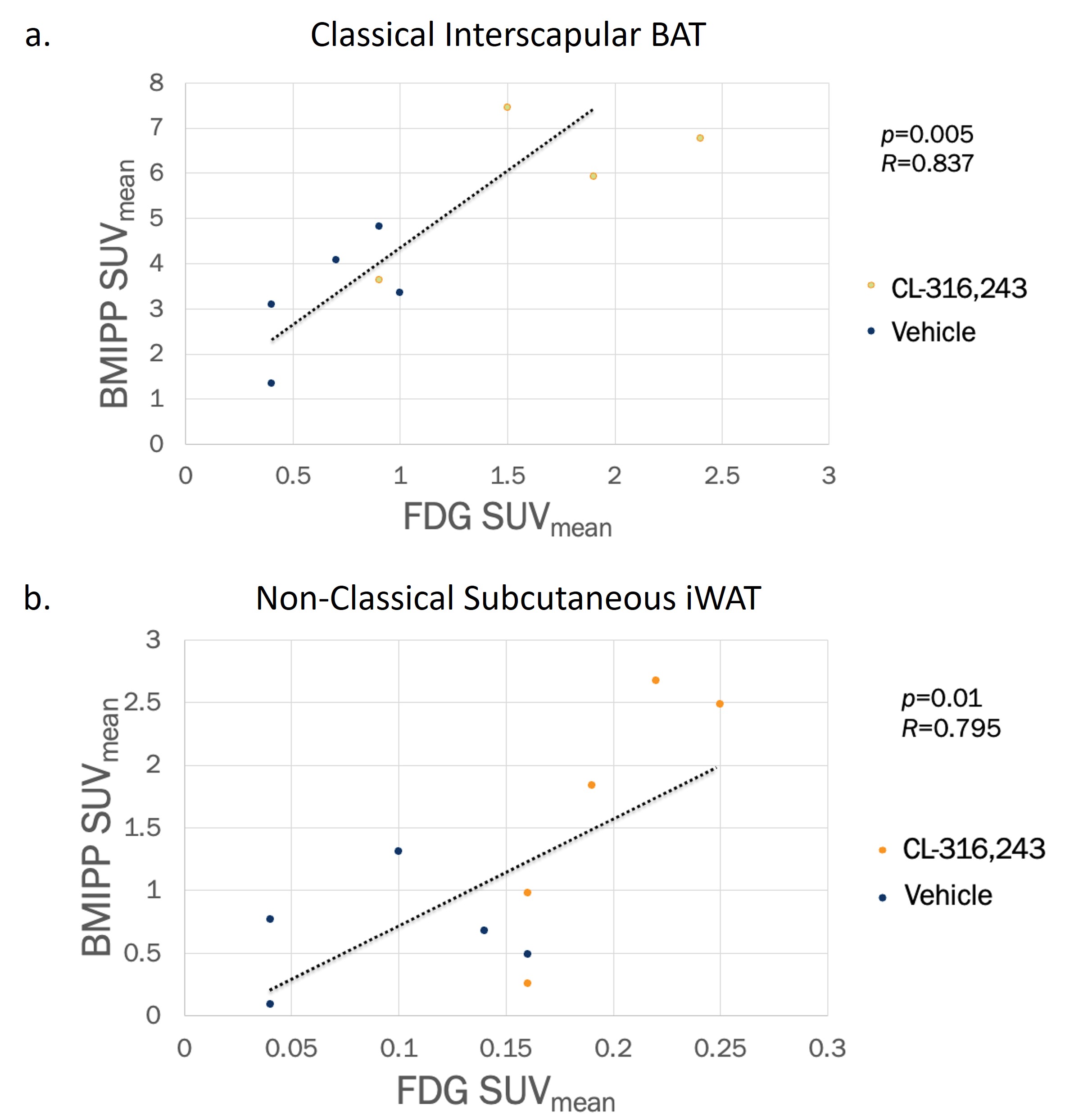

Supplement: Supplementary file 1 [file ijms-23-04880-s001.zip › Figure S2 - BMIPP and FDG Correlations.jpg]
